# Pharmacological Challenges of Novel Psychoactive Substances in Patients Receiving Opioid Substitution Therapy

**DOI:** 10.1192/bjo.2026.11856

**Published:** 2026-06-30

**Authors:** Mohammad Ali

**Affiliations:** Addictions Recovery Community (ARC-MK), Milton Keynes, United Kingdom

## Abstract

**Aims:**

Novel psychoactive substances (NPS) present unpredictable pharmacodynamic and pharmacokinetic effects, which can complicate opioid substitution therapy (OST) and increase overdose risk. Understanding patterns of NPS use in OST patients is important for optimizing pharmacological management and harm reduction. This study aimed to identify the prevalence and types of NPS use among OST outpatients, describe associated pharmacological risks, and evaluate whether such use was documented in clinical notes.

**Methods:**

A retrospective pharmacological review was conducted in an addictions psychiatry outpatient clinic. Adults aged ≥18 years receiving methadone or buprenorphine were included. Patients were excluded if clinical records were incomplete or if acute intoxication, withdrawal, or cognitive impairment precluded accurate reporting. Data were extracted from patient self-report, toxicology results, and clinical notes, focusing on type of NPS, concurrent substance use, OST dose, and documented adverse events such as sedation, withdrawal, or toxicity. Descriptive analyses summarized prevalence, pharmacological profiles, and documentation.

**Results:**

Among 120 patients (methadone n=75, buprenorphine n=45), 32 patients (27%) reported NPS use, most commonly synthetic cannabinoids (n=16) and cathinone derivatives (n=10). Polysubstance use occurred in 60% of NPS users versus 40% of non-users. Pharmacological complications were reported in 15 NPS users (47%), including enhanced sedation, withdrawal exacerbation, and increased craving. Notably, 23 of 32 patients (72%) had no prior documentation of NPS use in clinical notes, highlighting under-recognition of potential pharmacological risks.

**Conclusion:**

NPS use is common in OST patients and presents significant pharmacological challenges, particularly in the context of polysubstance use. Systematic screening and documentation of NPS exposure is feasible and essential for safe OST management, dose adjustment considerations, and prevention of adverse pharmacological interactions.

